# Bone Marrow Stem Cell Contribution to Pulmonary Homeostasis and Disease

**DOI:** 10.4172/2329-8820.1000162

**Published:** 2015-09-11

**Authors:** Lindsay T McDonald, Amanda C LaRue

**Affiliations:** 1 Research Services, Ralph H Johnson VAMC, Charleston, SC, USA; 2 Department of Pathology and Laboratory Medicine, Medical University of South Carolina, Charleston, SC, USA; 3 Hollings Cancer Center, Medical University of South Carolina, Charleston, SC, USA

**Keywords:** Bone marrow stem cells, Mesenchymal stem cells, Hematopoietic stem cells, Pulmonary homeostasis, Tissue regeneration

## Abstract

The understanding of bone marrow stem cell plasticity and contribution of bone marrow stem cells to pathophysiology is evolving with the advent of innovative technologies. Recent data has led to new mechanistic insights in the field of mesenchymal stem cell (MSC) research, and an increased appreciation for the plasticity of the hematopoietic stem cell (HSC). In this review, we discuss current research examining the origin of pulmonary cell types from endogenous lung stem and progenitor cells as well as bone marrow-derived stem cells (MSCs and HSCs) and their contributions to lung homeostasis and pathology. We specifically highlight recent findings from our laboratory that demonstrate an HSC origin for pulmonary fibroblasts based on transplantation of a clonal population of cells derived from a single HSC. These findings demonstrate the importance of developing an understanding of the sources of effector cells in disease state. Finally, a perspective is given on the potential clinical implications of these studies and others addressing stem cell contributions to lung tissue homeostasis and pathology.

## Introduction

Chronic lung diseases are extremely debilitating and are associated with high morbidity and mortality. As such, many physicians and scientists have turned to stem cell based therapies in hopes of regeneration and cure for these diseases. Leaders in the field of stem cell research have adopted the idea that the bone marrow harbors two stem cell types, the mesenchymal stem cell (MSC) and the hematopoietic stem cell (HSC). Classically, mesenchymal cell types such as tissue fibroblasts, adipocytes, osteoblasts, and chondrocytes were thought to originate exclusively from the MSC. HSCs have long been recognized as the origin for cells of the blood lineages such as B cells, T cells, and monocytes. This dichotomy was originally established based on embryonic lineage tracing studies and *in vitro* studies, which demonstrated that bone marrow stromal cells/MSCs could differentiate into cells of embryonic germ layer origin including bone, cartilage, muscle and adipose tissue. The HSC was established as a blood stem cell based on the discovery that rare single cells could give rise to myeloerythroid colonies in the spleens of irradiated transplant mice and that these cells had the capacity to self-renew [1,2]. While this distinction between the MSC and HSC potential persists, technologies have improved and cutting edge techniques have become available, such as single cell RNA isolation and analysis, elaborate lineage tracing methodologies, and improved scanning and imaging instruments. These innovative technologies have led to advances in the field of stem cell biology and have resulted in new questions regarding the lineage and plasticity of bone marrow populations. They have also led to an increased understanding of the contribution of specific populations of stem cells and their progenitors in the pathophysiology directly relevant to lung tissue regeneration and healing. Based on this, several clinical trials have been initiated examining the potential of bone marrow stem cells in lung disease [3,4]. Current experimental efforts rely mainly on MSC-based therapies; however, recent data by our group [5,6] and others [7] suggests that HSC-based treatments may also have clinical applications in the treatment of lung disease [8].

## Bone Marrow Stem Cells: MSCs and HSCs

The term MSC was originally coined based on the ability of this bone marrow-derived population to give rise to multiple, mesenchymal tissue types. An MSC is defined as an adherent, fibroblastoid-like cell that has the capacity to differentiate into osteoblasts, adipocytes, and chondrocytes *in vitro* [9]. Despite the proposed minimal criteria to define MSCs, ongoing limitations in the study of MSCs include a lack of clearly defined and universally agreed upon MSC markers, as well as lack of engraftment *in vivo* [9]. These limitations have largely confined the evidence for pluripotency to *in vitro* assays and have consequently led to an evolving nomenclature for the MSC [10]. While early *in vitro* evidence suggested a stem-like phenotype of MSCs, data to directly demonstrate stem cell capabilities has been lacking, therefore the term MSC was modified to describe this population as “multipotent stromal cells” [10]. Later, the identification of shared marker expression between MSCs and pericytes, which had multipotent differentiation capacity *in vitro* [11], and the finding that MSCs localize near the vasculature led to the theory that the MSC may be more closely related to, or may in fact, be a pericyte or a sub-population within the pericyte population [11,12]. Further confounding our understanding of MSCs and the MSC/pericyte relationship, our group [13] and others [14] have demonstrated an HSC origin for pericytes, suggesting that the MSC may actually be an intermediate phenotype between the two cell types. Regardless of this relationship, with respect to function, it has been suggested that rather than direct differentiation, the ability of MSCs to contribute to tissue repair may more significantly reflect their ability to produce soluble factors that alter the tissue microenvironment [15]. It is proposed that, in response to signals from the surrounding tissue, the MSC/pericyte becomes activated to promote a regenerative environment supporting differentiation of cell types such as fibroblasts, chondrocytes, and osteoblasts [16,17]. As further indication of the evolving definition [18,19] and increased understanding of the nature of MSCs, Caplan, a premier investigator in the field of MSC research, has hypothesized that the MSC may in fact be more aptly termed a “medicinal signaling cell” [17]. This term reflects the ability of the MSC to secrete bioactive molecules that act to support a regenerative microenvironment [17]. If the primary function of the MSC truly lies in its signaling function, then the identity of the bone marrow stem cell(s) that actually differentiates into mesenchymal cell types remains unclear.

Emerging lineage tracing studies have also informed our understanding of HSC plasticity such that the HSC is no longer thought to exclusively give rise to cells of the blood lineages. An HSC is well defined based on a distinct set of cell surface markers, and in transplantation studies, the stem identity of these cells can be confirmed based on hematopoietic reconstitution. Thus, the ability to clearly define the HSC based on both marker expression and functional assay has allowed for direct lineage tracing studies. These studies have demonstrated that the HSC gives rise to multiple tissue cell types including, pericytes, adipocytes, chondrocytes, osteoblasts, osteocytes, glomerular mesangial cells, hepatic stellate cells, fibrocytes, lung fibroblasts, and other tissue cell types [6,13,14,20–26]. While evidence is mounting for increased plasticity of the HSC, the topic is still debated due to conflicting reports [27,28]. The loss of hematopoietic markers such as the pan leukocyte marker, CD45, in many differentiated cellular populations has led to challenges in tracing cells from origin to maturity without the aid of additional surface markers, genetic markers, or fluorescent labels. In addition, the relative rarity of the HSC-derived populations in normal non-hematopoietic tissues, questions regarding cell fusion events, and the challenges of achieving a high level of engraftment for direct lineage tracing studies have confounded ongoing debates regarding HSC potential. Despite this, HSC based therapies have been a standard of care for over 60 years for hematological malignancies and correction of genetic abnormalities [29]. However, with regard to non-hematologic treatment and regeneration studies, despite the growing evidence for an HSC origin of tissue cell types, the utility of the HSC has been largely unexplored due to adherence to the classical definition of an HSC exclusively giving rise to blood cell types. Therefore, while research has begun to redefine the identity, fate, and roles of bone marrow stem cell populations, continued progress is needed towards expansion of the clinical application of MSC and HSC based therapeutics [29].

## Lung Homeostasis

Maintaining a homeostatic balance is an active process in the lung due to the constant exposure to the external environment. In this process, a delicate equilibrium between immune surveillance, inflammation, cellular activation/reaction, and cellular turnover must be achieved. Given this, a high threshold for reactive response is set in order to prevent an excessive response to daily exposures, insults, and minor injury. Despite the continual maintenance required to achieve this balance, the identification of a single, protective, pluripotent lung stem cell has been elusive, although several studies have indicated the presence of endogenous stem/progenitor populations in the lung. For example, basal cells found in the mouse trachea were shown to have the ability to give rise to Clara cells (club cells) and ciliated cells in the lung during postnatal growth and in the adult in steady state and in injury repair [30]. In addition, Clara cells in the proximal airway were shown to give rise to ciliated cells during epithelial homeostasis [31]. Bronchioalveolar stem cells (BASCs) have also been identified that express both Clara cell secretory protein and prosurfactant protein C and participate in lung homeostasis [32]. In the distal lung epithelium, type II pneumocytes have been shown to exhibit plasticity, through lineage tracing studies demonstrating their ability to contribute to the slow turnover of the alveolar epithelium in homeostasis [33]. Further, resident lung mesenchymal stromal cells (LMSCs) have also been identified that share commonalities with bone marrow-derived MSCs, although their roles in homeostasis are not yet fully defined [34–36]. While these studies demonstrate the presence and contribution of resident populations in maintenance of lung steady state, the role of bone marrow-derived stem cells in lung homeostasis is not well studied with the exception of immune cells that participate in routine immune surveillance and response to acute exposures. Although lineage-tracing studies have demonstrated limited contribution of bone marrow-derived stem cell populations to lung [6,37–39], fully understanding the contribution of bone marrow-derived cells to lung homeostasis has been challenging due to lack of experimental models. Further, several studies suggest the requirement of a reactive state (i.e., acute injury) in order to induce bone marrow stem cell-derived cell recruitment to the lung [37,38]. Even in the case of host defense, macrophage clearance of inhaled debris does not appear to be required until challenged by infection, chemical or physical damage, or other acute injury in a reactive homeostatic response [40]. Together, these studies suggest that homeostasis is maintained predominantly through resident cell populations in the lung, with bone marrow-derived cells potentially participating in the reactive response necessary to return to a homeostatic state following acute injury.

## Lung Disease

In contrast to homeostasis wherein resident cell populations appear to be primarily responsible for maintenance and cell turnover, studies suggest that in chronic pathological conditions, bone marrow-derived populations become more prominent contributors. A unifying feature amongst many chronic lung pathologies is the destruction of normal lung architecture, and such is the case for chronic obstructive pulmonary disease (COPD), asthma, bronchopulmonary dysplasia, and idiopathic pulmonary fibrosis. In diseases such as these, the rapid turnover and increased recruitment of cells to the injury site has aided the study of bone marrow-derived stem cells in lung, suggesting also that bone marrow derived cells are actively recruited as a result of tissue injury [41]. Analysis following stem cell transplantation in humans has shown the presence of bone marrow-derived cells in lung based on sex mismatched transplant [42–44], with evidence demonstrating increased contribution of bone marrow-derived cells associated with cases of severe, chronic injury [42–44]. Additionally, pre-clinical studies in fibrotic lung disease models have demonstrated the presence of collagen I expressing bone marrow-derived cells in lung [45,46]. Bone marrow-derived type I pneumocytes have also been detected in lungs following bleomycin-induced lung injury [47] and type II pneumocytes of bone marrow origin were detected following radiation induced injury [48]. Studies have also demonstrated that contribution of bone-marrow derived cells to lung epithelium is dependent on degree and type of damage induced [48]. These studies suggest that bone marrow contribution to lung tissue is increased by cellular damage that results in elevated cell turnover. While many organs have been demonstrated to possess the capacity to heal and/or regenerate, the lung appears to have a limited capacity for regeneration/restoration of normal architecture, despite the potential contribution of endogenous and bone marrow-derived stem and progenitor cells to pulmonary cell types.

### Fibroblasts as effectors of lung disease

The loss of normal tissue architecture in lung disease is a process that is predominantly mediated by the balance of turnover and deposition of extracellular matrix and the primary effector cells in these processes are thought to be the fibroblasts. In particular, fibrosis has been shown to be reversible in many organs based on targeting fibroblast populations; however, there is currently a lack of evidence for this reversibility in lung. One complicating factor is that the fibroblasts in lung pathologies are thought to have multiple origins, including resident fibroblasts and those from extra-pulmonary sources [49]. As discussed above, several studies demonstrate a bone marrow contribution of cells in models of lung pathology; however, the stem cell from which these cells are derived is not clearly defined. This is particularly evident in studies of fibroblasts and their bone marrow-derived precursors, fibrocytes, in lung. Fibrocytes are fibroblastic precursor cells that are found in the circulation and in tissues at sites of wound healing [50] that, due to their circulating nature and expression of certain hematopoietic markers, are thought to be of hematopoietic origin [22,24,51]. Based on their similarity to both fibroblasts and MSCs, many others consider fibroblasts and/or fibrocytes to either be derived from MSCs or to be closely related to the MSC [52,53]. While still debated, the origin of the fibrocyte [54] and the lung fibroblast is of particular significance given that understanding the sources of these effector cells may allow for enhanced targeting of this population in many pulmonary diseases that share the a commonality in their altered matrix balance/turnover and eventual loss of normal lung architecture.

### An HSC origin for fibroblasts

Recent research in our laboratory, has demonstrated contribution of the HSC to the lung fibroblast population and identified an HSC-derived circulating fibroblast precursor (CFP) population in both the blood and lungs [5,6]. Previous studies in our laboratory have suggested that the CFP is derived along the monocyte lineage and contains the fibrocyte population, supporting an HSC origin for fibrocytes/fibroblasts [5,7]. These studies were based on a direct lineage tracing model wherein a single enhanced green fluorescent protein-expressing (EGFP^+^) HSC is subjected to short-term culture *in vitro* to derive a clonal cell population that is then transplanted into lethally irradiated recipient mouse allowing for identification of the HSC-derived populations based on expression of EGFP. Using this clonal cell transplantation model, we also demonstrated the persistence of the HSC contribution to the lung fibroblast population, with HSC-derived fibroblasts present up to one year post transplant [6]. This was in agreement with data by Krause et al. which demonstrated persistence of HSC-derived cells, including those in the bronchii and alveoli, in lung for 11 months post-transplant [37]. Further, our findings suggested a functional role for HSC-derived fibroblasts and circulating fibroblast precursors in lung as matrix producing and matrix sensing cells based on expression of collagen I and the collagen receptor discoidin domain receptor-2 (DDR2) [6]. The presence of these circulating fibroblast precursors in both blood and lungs, suggest an ability of the HSC-derived population to continuously home from the bone marrow through the blood and to the lung [6]. Together, these findings support an underappreciated contribution of and role for the HSC in lung and indicate that HSCs may contribute significantly to lung pathologies.

## Clinical Implications and Perspectives

Increasing appreciation for the plasticity of the HSC and the potential of a MSC intermediate may lead to exciting new therapeutic applications and possibilities in the clinic. Current novel therapeutics for multiple diseases have employed MSC-based therapies, as well as specialized HSC transplantation studies [29]. An understanding of the body as a complex signaling machine with the bone marrow being both a source of cellular effectors and a protective niche/storage site aligns with a holistic clinical approach. In the case of respiratory disease, treating the lungs and the chief clinical complaint may not be sufficient to cure the disease, perhaps because the primary source of the effector cells has not been corrected. From a therapeutic perspective, exclusion of a critical contributing HSC-derived population in previous studies also leaves many stones unturned in the search for disease initiating events, especially in the case of idiopathic pulmonary fibrosis, perhaps the most insidious of the lung diseases. We recognize the bone marrow acts as a protective niche for critical stem cells in the body, without which one cannot survive long term. In the case of pathological conditions, such as cancer, aberrant stem cell/tumor initiating cells have also been reported to reside in the bone marrow, where they are protected. Given this protective environment, it is also possible that an HSC that has been altered by exposure to some initiating pathology may persist, giving rise to altered production of circulating cells that cycle through the activated lung environment and in turn, transmits damage signals back to the bone marrow niche. Thus, in lung pathologies such as pulmonary fibrosis where the loss of normal lung architecture is a defining feature, organ transplantation alone may not be sufficient to re-program fibroblast precursors or other effector/progenitor cells originating from the bone marrow. In this way, lung transplantation alone may result in the preservation of a safe haven for cells primed to contribute to a pro-fibrotic lung environment. Therefore, an adjunct treatment centered on reversing the activated phenotype or skewing the differentiation of stem-cell derived populations away from a fibroblastic/pro-fibrotic pathway towards a regenerative/homeostatic pathway may be beneficial for patients receiving lung transplants.

**Figure 1:**
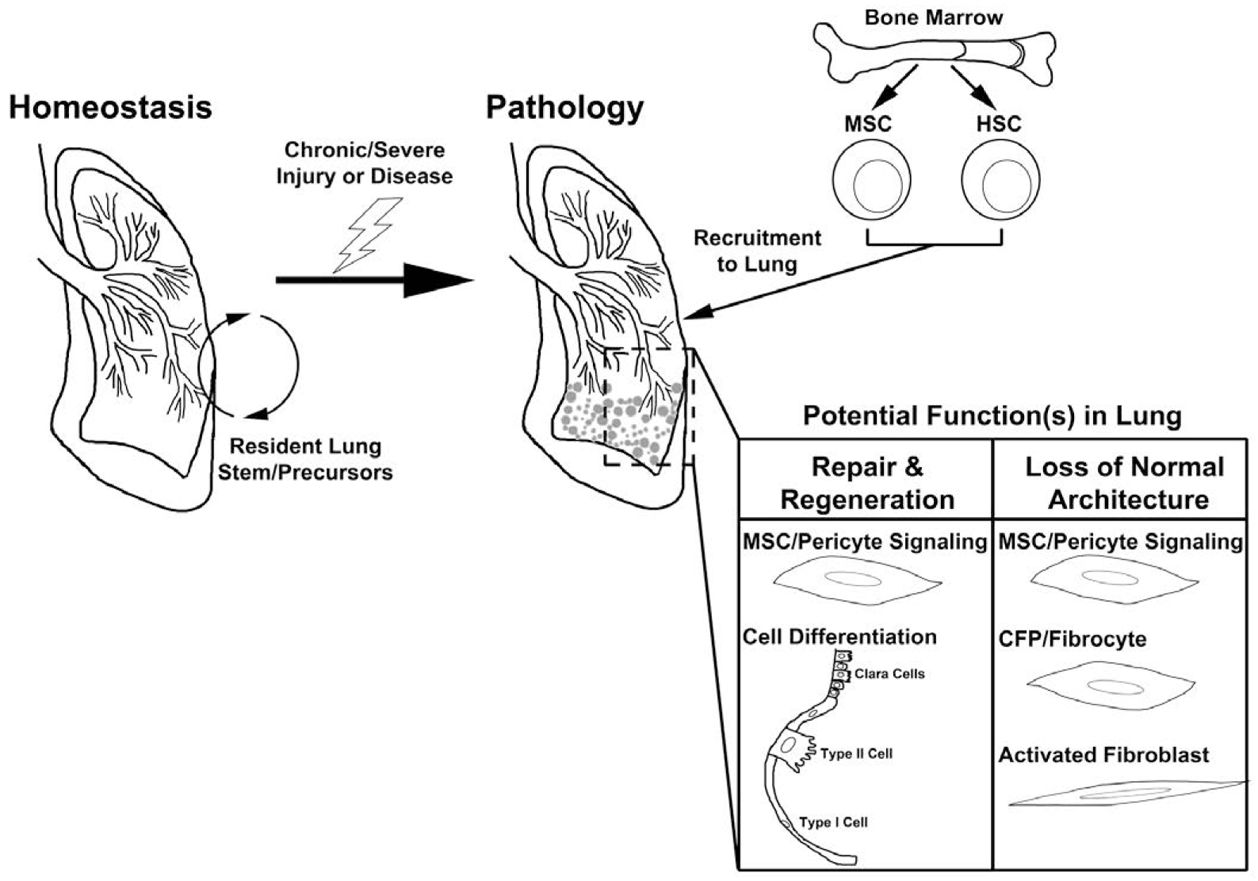
Bone marrow-derived stem cells in lung pathophysiology. Studies suggest that lung homeostasis is maintained predominantly by resident stem and progenitor populations, with the exception of bone marrow-derived immune cells that are responsible for tissue surveillance. In the case of chronic injury, severe damage, or disease, bone marrow-derived MSCs and HSCs are recruited to the lung where they play multiple potential roles. These cells have been suggested to contribute to lung tissue repair and regeneration as well as perpetuation of disease by contributing to and/or driving loss of lung architecture. Elucidation of the contributions of specific stem cell/progenitor populations in each of these processes is essential towards harnessing their potential for therapeutic purposes.

With respect to regenerative applications of bone marrow-derived stem cells in lung diseases, clinical trials have predominantly focused on the impact of MSC-based therapeutics. While some efficacy has been demonstrated in several lung diseases including pulmonary fibrosis and COPD, these studies have been hampered by a lack of high level of engraftment of the MSC, and the mechanism(s) behind the beneficial effects are unclear. This may suggest that, as Caplan’s group has indicated [17], the MSC is acting as a signaling cell indicating to the surrounding cells, and perhaps signaling back to the stem populations in the bone marrow (i.e., the HSC), that regeneration and restoration of homeostasis is required. This idea harkens back to early stem cell studies that demonstrated that the MSC may act as a supportive cell for the HSC in the bone marrow niche [55]. In this way, the MSC may critically signal for regeneration, whereas the HSC may be the regenerative/reparative cellular source in the disease state. This idea is supported by increasing evidence of HSC engraftment and plasticity in multiple organs [25,26,37]. Importantly, the MSC or HSC-derived populations may also have utility as drug delivery agents to promote restoration and/or regeneration due to their capacity to home to and incorporate into the lung tissue, as well as through their direct cross-talk with the surrounding microenvironment and cellular milieu. Thus, in lung pathophysiology, both the MSC and HSC may play critical roles in promoting homeostasis, in affecting disease, and in potential healing and tissue regeneration (Figure 1).

It is critical that, as knowledge in the field of stem cell biology progresses, so too does our willingness to challenge dogma that may restrict progress. Likely, there is a place for both MSCs and HSCs in the ultimate cure of pulmonary disease as well as other diseases, although the role(s) of each of these stem cells may not be as initially thought. An increasing understanding of all origins of the lung cell populations and the cross-talk and environmental signals that regulate their differentiation and function may, therefore, lead to discovery of previously unrecognized initiating events and may uncover exciting new therapeutic opportunities for lung diseases. We are just beginning to appreciate the plasticity of bone marrow stem cell and signaling cell populations. Recognizing that both the MSC and HSC may be key components towards identifying more effective treatments and adjunct therapies or alternatives to lung transplantation is necessary to improve patient prognosis, quality of life, and survival rates.
